# Production of extracellular α-amylase by single-stage steady-state continuous cultures of *Candida wangnamkhiaoensis* in an airlift bioreactor

**DOI:** 10.1371/journal.pone.0264734

**Published:** 2022-03-01

**Authors:** Griselda Ma. Chávez-Camarillo, Perla Vianey Lopez-Nuñez, Raziel Arturo Jiménez-Nava, Erick Aranda-García, Eliseo Cristiani-Urbina

**Affiliations:** 1 Instituto Politécnico Nacional, Escuela Nacional de Ciencias Biológicas, Departamento de Microbiología, Prolongación de Carpio y Plan de Ayala s/n, Colonia Casco de Santo Tomás, Ciudad de México, CP, México; 2 Instituto Politécnico Nacional, Escuela Nacional de Ciencias Biológicas, Departamento de Ingeniería Bioquímica, Unidad Profesional Adolfo López Mateos, Ciudad de México, CP, México; Konkuk University, REPUBLIC OF KOREA

## Abstract

The kinetics of growth and α-amylase production of a novel *Candida wangnamkhiaoensis* yeast strain were studied in single-stage steady-state continuous cultures. This was performed in a split-cylinder internal-loop airlift bioreactor, using a variety of carbon sources as fermentation substrates. Results showed that the steady-state yields of cell mass from carbohydrates were practically constant for the range of dilution rates assayed, equaling 0.535 ± 0.030, 0.456 ± 0.033, and 0.491 ± 0.035 g biomass/g carbohydrate, when glucose, maltose, and starch, respectively were used as carbon sources. No α-amylase activity was detected when glucose was used as the carbon source in the influent medium, indicating that α-amylase synthesis of *C*. *wangnamkhiaoensis* is catabolically repressed by glucose. Contrastingly, maltose and starch induce synthesis of α-amylase in *C*. *wangnamkhiaoensis*, with starch being the best α-amylase inducer. The highest α-amylase volumetric and specific activities (58400 ± 800 U/L and 16900 ± 200 U/g biomass, respectively), and productivities (14000 ± 200 U/L·h and 4050 ± 60 U/g biomass·h, respectively) were achieved at a dilution rate of 0.24 h^-1^ using starch as the carbon source. In conclusion, single-stage steady-state continuous culture in an airlift bioreactor represents a powerful tool, both for studying the regulatory mechanisms of α-amylase synthesis by *C*. *wangnamkhiaoensis* and for α-amylase production. Furthermore, results showed that *C*. *wangnamkhiaoensis* represents a potential yeast species for the biotechnological production of α-amylase, which can be used for the saccharification of starch. This offers an attractive renewable resource for the production of biofuels (particularly bioethanol), representing an alternative to fossil fuels with reduced cost of substrates.

## 1. Introduction

Amylases (α, β, and γ) are among the most versatile and important families of enzymes that have numerous biotechnological applications [1,2]. Among them, α-amylases (α-1,4-glucan-4-glucanohydrolase; E.C. 3.2.1.1.) are extensively used in several industries. For instance, food, brewery, syrups, textile, detergent, pulp, paper, pharmaceutical, environmental, biofuel, healthcare, clinical, and analytical chemistry industries, among others [1,3,4]. Nowadays, α-amylases hold the major world market share of enzymes, accounting for approximately 25–30% of the world´s enzyme production [2,3].

α-Amylases catalyze the hydrolysis of starch by cleaving the internal α-1,4-glycosidic linkages to produce limit dextrin, short-chain oligosaccharides, maltotriose, maltose, and glucose [3]. Although α-amylases can be obtained from several sources, such as animals, plants, and microorganisms, most industrial applications use microbial α-amylases, mainly from bacteria and filamentous fungi [5].

Yeasts offer several advantages for the commercial production of amylases, including: their easier cultivation, higher specific growth rate and shorter generation time than filamentous fungi ensure more productivity. Because of their GRAS (generally regarded as safe) status, their products are safe for human consumption, and yeasts are commonly deemed superior to bacteria in terms of their extracellular products. Yeasts are also amenable to easy genetic modification. Enzymes-derived from yeast tend to be more economically relevant than enzymes derived from other sources [6–10].

α-Amylase production is not widespread among yeast species but is found principally among species of the genera *Candida*, *Cryptococcus*, *Lipomyces*, *Pichia*, *Saccharomycopsis*, and *Schwanniomyces* [4,6,7]. Amylolytic yeasts have garnered much attention in recent years because of their great potential to produce single-cell oils, single-cell proteins, ethanol, and alcoholic beverages from starchy substrates [6,8,11,12]. Furthermore, yeast amylases have properties that differ from those produced by other microorganisms, which make them potentially useful in several industries, including food, biofuels, clinical, pharmaceutical, analytical chemistry, and those involving environmental remediation [3,6,10].

Recently, Hernández-Montañez et al. [11] reported that only 1 of 385 yeast strains isolated from soil and foods was able to degrade starch. This yeast strain was genetically identified by sequencing the D1/D2 domain of the 26S rDNA gene as *Wickerhamia* sp. X-Fep. It is now designated as *Candida wangnamkhiaoensis* [13]. This yeast species was recently reported in the literature but has scarcely been studied. Therefore, its physiological and metabolic capacities, as well as its potential for use in biotechnological processes, are unknown. Our research group previously demonstrated that *C*. *wangnamkhiaoensis* can produce α-amylase and reported some biochemical and molecular properties of the enzyme [11]. We also demonstrated that *C*. *wangnamkhiaoensis* α-amylase is capable of extensive starch hydrolysis and exhibits an elevated capacity for starch saccharification. In the light of this information, further research to determine the potential of *C*. *wangnamkhiaoensis* as an α-amylase producer will be of great value.

To optimize the α-amylase production process of *C*. *wangnamkhiaoensis*, it is essential to investigate the mechanisms underlying yeast growth and enzyme synthesis.

The continuous and uncontrollable changes in the environmental conditions during microbial culture growth lead to continuous alteration of the culture phenotype. This makes the results of batch studies on regulatory mechanisms of enzyme synthesis difficult to interpret. Furthermore, it is generally not possible to alter the growth environmental composition in batch cultivation without simultaneously changing the culture growth rate and consequently the culture physiology [14,15].

In contrast, the chemostat is a powerful tool for studying the physiology of microbial cultures because cell growth occurs under steady-state conditions. Here, the environmental growth conditions and consequently the culture phenotype, remain invariant throughout the cultivation period. Additionally, a continuous chemostat culture permits the alteration of environmental growth conditions independent of the specific growth rate of the microbial culture [14–16].

Continuous chemostat cultures have potential advantages for production purposes against batch cultures because the specific rate of microbial growth can be controlled. Additionally, long-term, higher volumetric and specific productivities, and higher yields of products can be achieved by manipulating the feed flow rate. This allows maintenance of low metabolic product concentrations in the chemostat to reduce growth inhibition [17,18]. Furthermore, compared to batch cultures, continuous chemostat cultures reduce time-consuming activities such as cleaning, filling, and sterilization of bioreactors, allow the use of smaller bioreactors, reduce capital investments, labor, production costs, and are easier to control at steady state [18,19].

Most studies on the production of yeast α-amylases have been performed in batch systems and the few studies available in the literature on amylolytic yeasts in continuous systems have focused on the production of biomass (unicellular protein) [20–23]. Furthermore, studies on amylolytic yeasts have been carried out in flasks or mechanically shaken bioreactors. To our knowledge, no studies have been reported using amylolytic yeasts grown in pneumatic bioreactors, such as in airlift bioreactors or bubble columns; these exhibit several economic and operational advantages over mechanically stirred bioreactors.

This study reports the production kinetics of α-amylase produced by *C*. *wangnamkhiaoensis* in single-stage steady-state continuous cultures in an airlift bioreactor, using different carbon and energy sources as fermentation substrates.

## 2. Materials and methods

### 2.1. Microorganism

A novel anamorphic yeast strain that was isolated from lemon and genetically identified as *Candida wangnamkhiaoensis* was used in this study. This yeast strain was acquired from the Microbial Culture Collection of the Industrial Microbiology Laboratory, Microbiology Department of the Biological Sciences National School, National Polytechnic Institute, Mexico City, Mexico. The *C*. *wangnamkhiaoensis* yeast strain was preserved on YPG (1% yeast extract, 1% casein peptone, and 2% glucose) agar slants and agar plates at 4°C.

### 2.2. Culture media composition

The culture medium used for the single-stage steady-state continuous culture experiments was made from Castañeda medium (0.375 g/L KH_2_PO_4_, 0.625 g/L dibasic ammonium citrate, 1 g/L yeast extract, 0.275 g/L MgSO_4_·7H_2_O, 0.25 g/L NaCl, and 0.375 g/L Na_2_CO_3_) [24] supplemented with either 20 g/L of glucose, maltose, or soluble starch. The culture media were sterilized in an autoclave at 121°C for 15 min. The pH of the culture medium was 6.0 ± 0.1. Analytical grade reagents provided by BD Bioxon (Mexico) and JT Baker (Mexico) were employed for the preparation of preservation and culture media.

### 2.3. Inoculum preparation

The inocula of *C*. *wangnamkhiaoensis* were grown in 200 mL of Castañeda’s culture medium supplemented with 20 g/L glucose, maltose, or soluble starch in 1000-mL Erlenmeyer flasks in a water bath shaker (Cole-Parmer, Inc., Vernon Hills, IL, USA) at 28 ± 2°C and 150 rpm for 15 h. The resulting yeast cell suspension was used as inoculum in subsequent continuous culture experiments performed in a pneumatic bioreactor.

### 2.4. Split-cylinder internal-loop airlift bioreactor

*Candida wangnamkhiaoensis* is an anamorphic yeast [13], highly susceptible to shear stress occurring in mechanically agitated bioreactors. This causes damage to pseudo-mycelial yeast cells and reduces α-amylase production. Airlift bioreactors are known to be suitable for shear stress-sensitive microorganisms, prone to physical damage by mechanical agitation or fluid turbulence. In addition, airlift bioreactors consume lower amounts of energy than mechanically stirred reactors, and are considered one of the most economic designs for aerobic industrial production systems [25,26]. Therefore, to minimize damage to *C*. *wangnamkhiaoensis* yeast cells, increase α-amylase production, and reduce production costs, an airlift bioreactor was used in the present study.

After performing a comprehensive literature search, no information was found on α-amylase production by yeasts in airlift bioreactors, so this is the first study on this subject matter.

**Fig 1:**
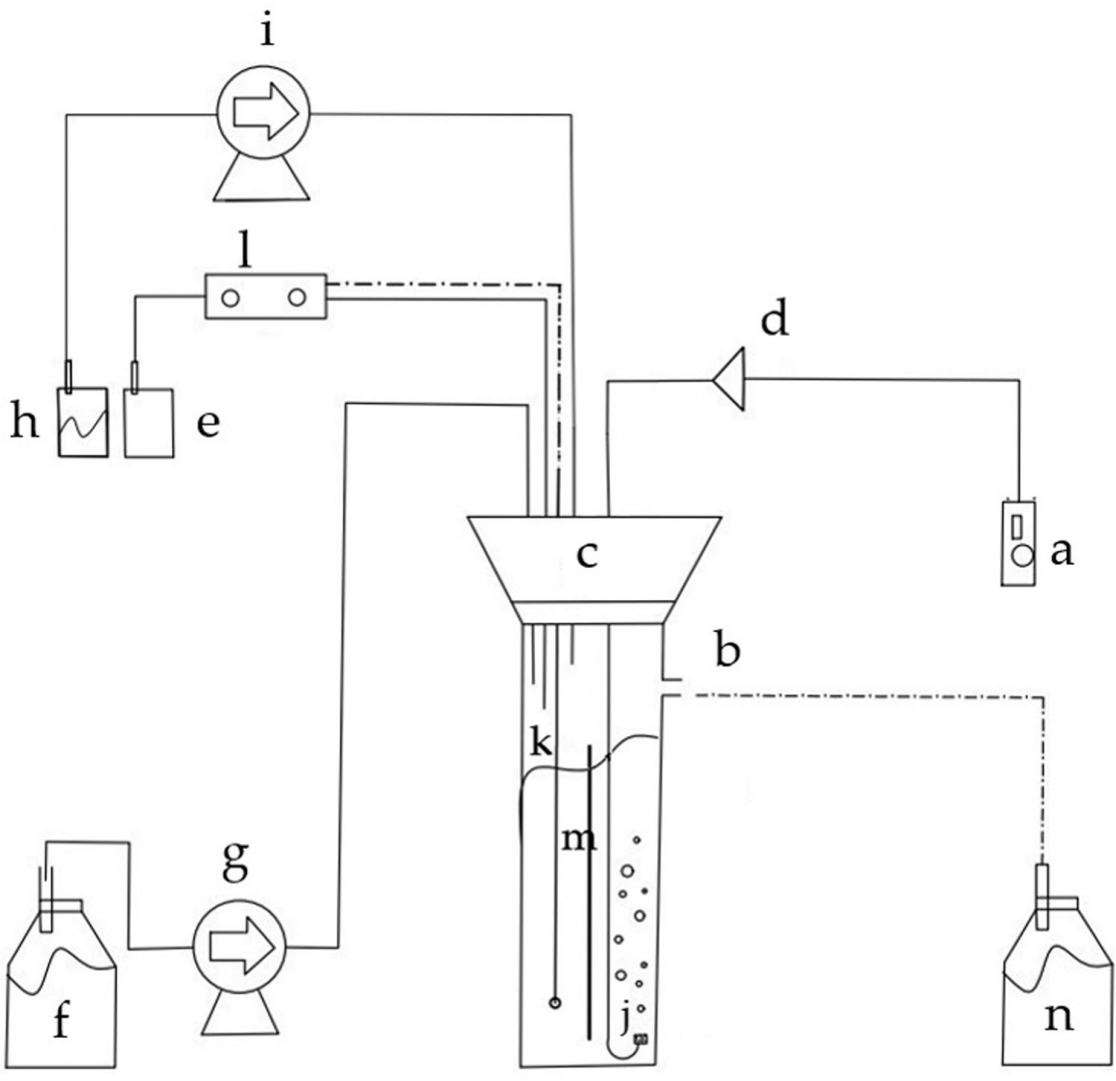
Schematic representation of the split-cylinder internal-loop airlift bioreactor. a) air pump; b) outflow effluent duct; c) silicone stopper; d) air-in filter; e) acid/alkali solution reservoir; f) culture medium reservoir; g) and i) peristaltic pumps; h) antifoam solution reservoir; j) gas diffuser; k) pH electrode; l) pH controller; m) baffle; n) effluent reservoir.

Single-stage steady-state continuous culturing was performed in a split-cylinder internal-loop airlift bioreactor with a liquid operating volume of 1.8 L. Fig 1 displays a schematic diagram of the airlift bioreactor. The cylindrical column and base of the bioreactor were made of borosilicate glass. The column bioreactor had a height of 50 cm and an inner diameter of 8 cm. Inside the column, there was a centered stainless-steel baffle, to divide the bioreactor column into two equal vertical parts. One part is the riser and the other is the downcomer. Therefore, the cross-sectional area ratio of the riser-to-downcomer is 1.0. The bottom and top clearances were 2 and 4 cm, respectively. The column body and the baffle side edges are so tightly joined that even liquids and air cannot pass along their juncture. The cylindrical column also had a port through which the culture samples were collected.

The upper part of the column has a silicone stopper with ports for the addition of sterile air, alkali, acid, inoculant, and to introduce pH and oxygen electrodes. Sterile air was sparged into the bottom of the bioreactor through a porous diffuser attached to the air duct outlet. The airflow rate was controlled using a pressure-regulating valve and measured using a calibrated rotameter. Sterile air was supplied to the bioreactor at a flow rate of 1.8 L/min (1 vvm).

### 2.5. Kinetics of *C*. *wangnamkhiaoensis* α-amylase production in single-stage steady-state continuous cultures

The effects of glucose, soluble starch, and maltose on α-amylase production were investigated in single-stage continuous cultures in an airlift bioreactor at pH 6.0 ± 0.1, 28 ± 2°C and 1 vvm of sterile air.

Single-stage continuous cultures were initiated with a batch culture and allowed to run for 20 h. Afterwards, sterile culture medium containing 20 g/L glucose, maltose, or soluble starch was continuously supplied into the airlift bioreactor at a known flow rate using a peristaltic pump (Masterflex L/S, USA), until the yeast biomass concentration, residual carbohydrate concentration, and α-amylase activity did not vary significantly with respect to time (p > 0.05). This occurred approximately at 3–4 liquid residence times, indicating that a steady state had been reached.

Different culture medium flow rates were assayed, thus assessing different dilution rates (D), which are equal to the specific growth rate of the yeast at steady state. Culture samples collected at steady state were analyzed immediately to quantify biomass and residual carbohydrate concentrations, and α-amylase activity.

### 2.6. Analytical methods

The yeast biomass concentration was measured gravimetrically as the cell dry weight. Yeast cells from 5 mL of culture were harvested by filtration on GF/A (Whatman) filters, washed three times with deionized water, and oven-dried at 90°C to reach a constant weight. The obtained filtrates were used to quantify the residual carbohydrate concentration and α-amylase activity.

Glucose, maltose, and starch concentrations were measured by enzymatic methods using glucose (GO), maltose, and starch (GO/P) assay kits (Sigma-Aldrich, St. Louis, MI, USA), according to the procedures outlined by the manufacturer.

α-Amylase activity was measured using the starch azure assay (S7629; Sigma-Aldrich, St. Louis, MI, USA), following the methodology described by Chávez-Camarillo et al. [27]. One unit of α-amylase activity (U) was defined as an increase in one unit of absorbance at 595 nm per hour at 50°C.

The α-amylase volumetric activity (P, U/L) was defined as the number of enzyme units per liter. Likewise, the specific activity of α-amylase or enzyme yield (Y_px_, U/g biomass) was expressed as the α-amylase volumetric activity per dry weight biomass concentration (X, g/L) of *C*. *wangnamkhiaoensis* yeast (P/X). The α-amylase volumetric productivity (Q_p_, U/L·h) was estimated as the product of the α-amylase volumetric activity (P) at the steady-state and operating dilution rate (D). Similarly, α-amylase specific productivity (q_p_, U/g biomass·h) was computed as the product of the steady-state α-amylase specific activity (Y_P/X_) and operating dilution rate (D) [28].

### 2.7. Statistical analysis

At least three independent continuous culture experiments were performed. GraphPad Prism software version 8.4 was used to perform the statistical analysis of the experimental data by means of one-way analysis of variance (ANOVA) with Tukey’s test and with a 95% confidence level (GraphPad Software, Inc., La Jolla, CA, USA).

## 3. Results and discussion

**Fig 2:**
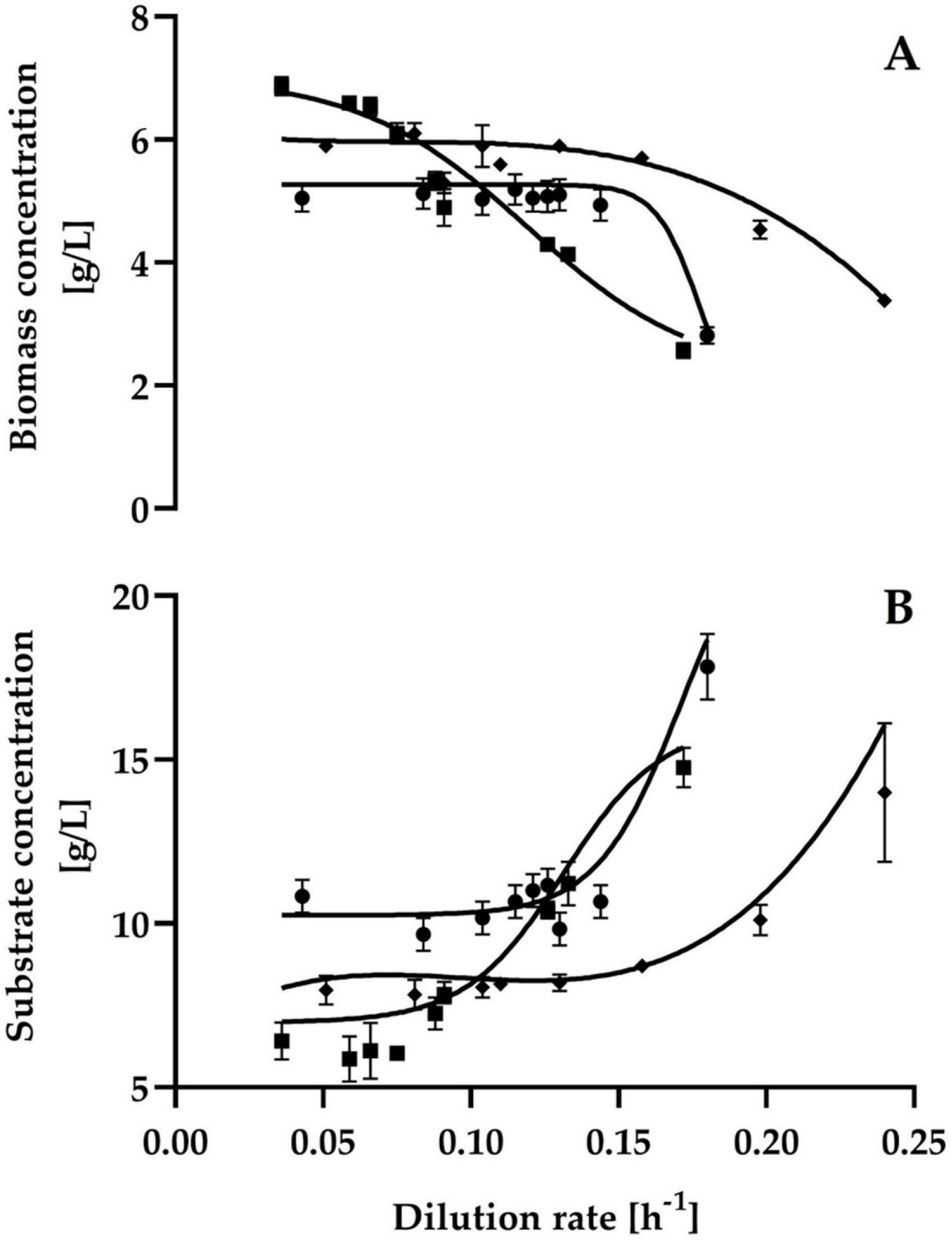
Steady-state biomass (A) and substrate (B) concentrations at different dilution rates by *C*. *wangnamkhiaoensis* in continuous culture. ●, glucose; ◼, maltose; ◆, starch.

The steady-state biomass and residual substrate concentrations in single-stage continuous cultures are shown in Fig 2. The growth and substrate consumption patterns were consistent.

The highest steady-state biomass concentration (6.87 ± 0.15 g/L) was achieved at a dilution rate of 0.036 h^-1^ using maltose as the carbon source in the feeding medium. However, at higher dilution rates (> 0.036 h^-1^), a continuously decreasing biomass concentration with an increasing dilution rate was observed (Fig 2A). In contrast, the biomass concentration at the steady-state was essentially constant in the range of dilution rates from 0.043 to 0.13 h^-1^ and from 0.051 to 0.13 h^-1^, when glucose (~ 5.09 ± 0.24 g/L) and soluble starch (~ 5.95 ± 0.18 g/L) were used as carbon sources, respectively. However, the biomass concentrations decreased at dilution rates higher than 0.13 h^-1^.

**Fig 3:**
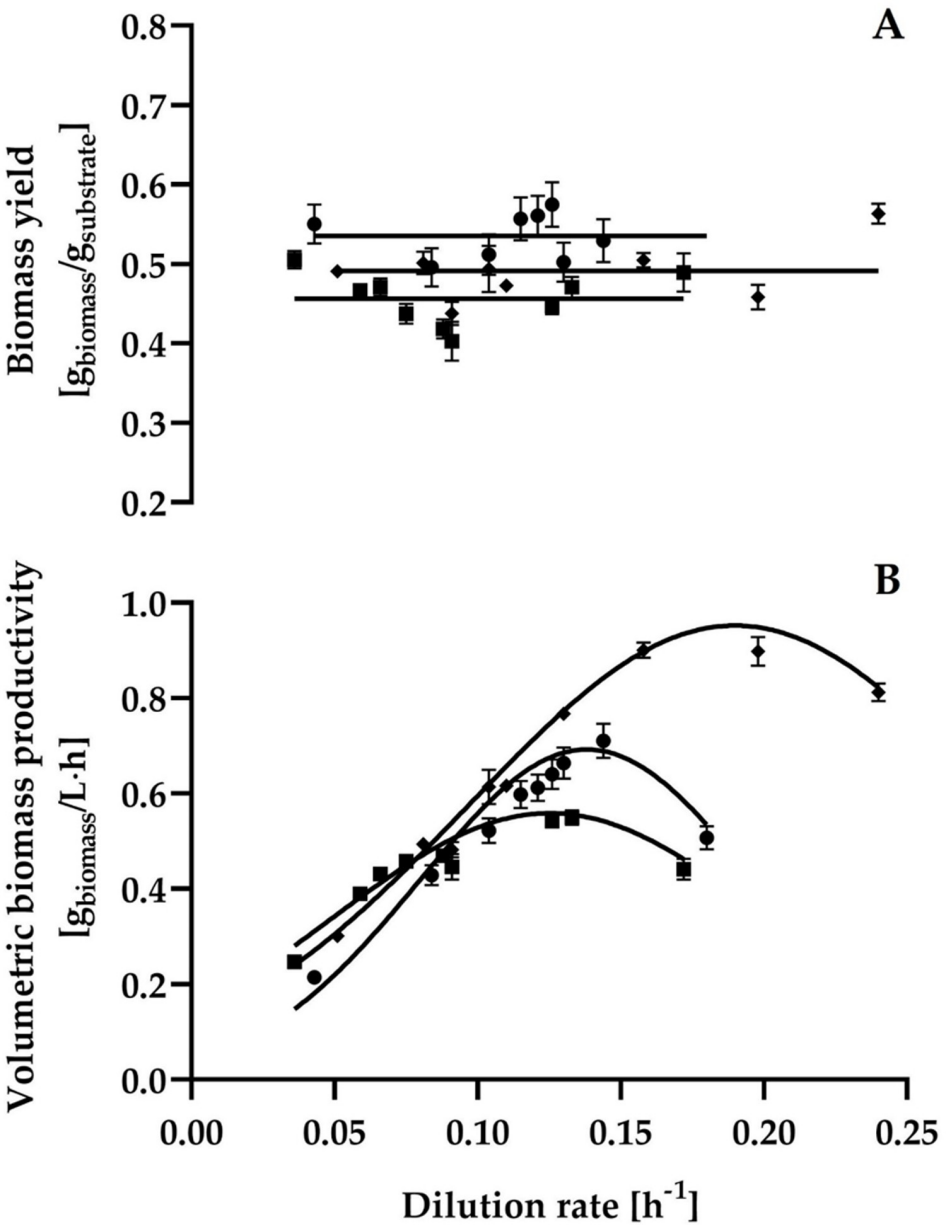
Steady-state biomass yield (A) and biomass productivity (B) at different dilution rates by *C*. *wangnamkhiaoensis* in continuous culture. ●, glucose; ◼, maltose; ◆, starch.

The steady-state yields of cell mass from carbohydrates (Y_xs_) were practically constant in the range of dilution rates that were examined (Fig 3A). When glucose, maltose, and starch were used as carbon sources, the mean cell yield values were 0.535 ± 0.030, 0.456 ± 0.033, and 0.491 ± 0.035 g biomass/g carbohydrate, respectively. A qualitatively similar behavior was reported for *Saccharomycopsis fibuligera* when this yeast was grown on a potato extract simulating processing blancher water [20,29]. Additionally, it has been reported that the *S*. *fibuligera* biomass yield from starch is approximately 0.55 g/g [29], which is slightly higher than that obtained in this study (0.491 ± 0.035 g/g).

The maximum levels of volumetric productivity of biomass were reached at dilution rates of 0.144, 0.133, and 0.158 h^-1^, with values of 0.71 ± 0.036, 0.55 ± 0.015, and 0.9 ± 0.016 g biomass/L·h, when glucose, maltose, and starch were used as substrates, respectively (Fig 3B).

**Fig 4:**
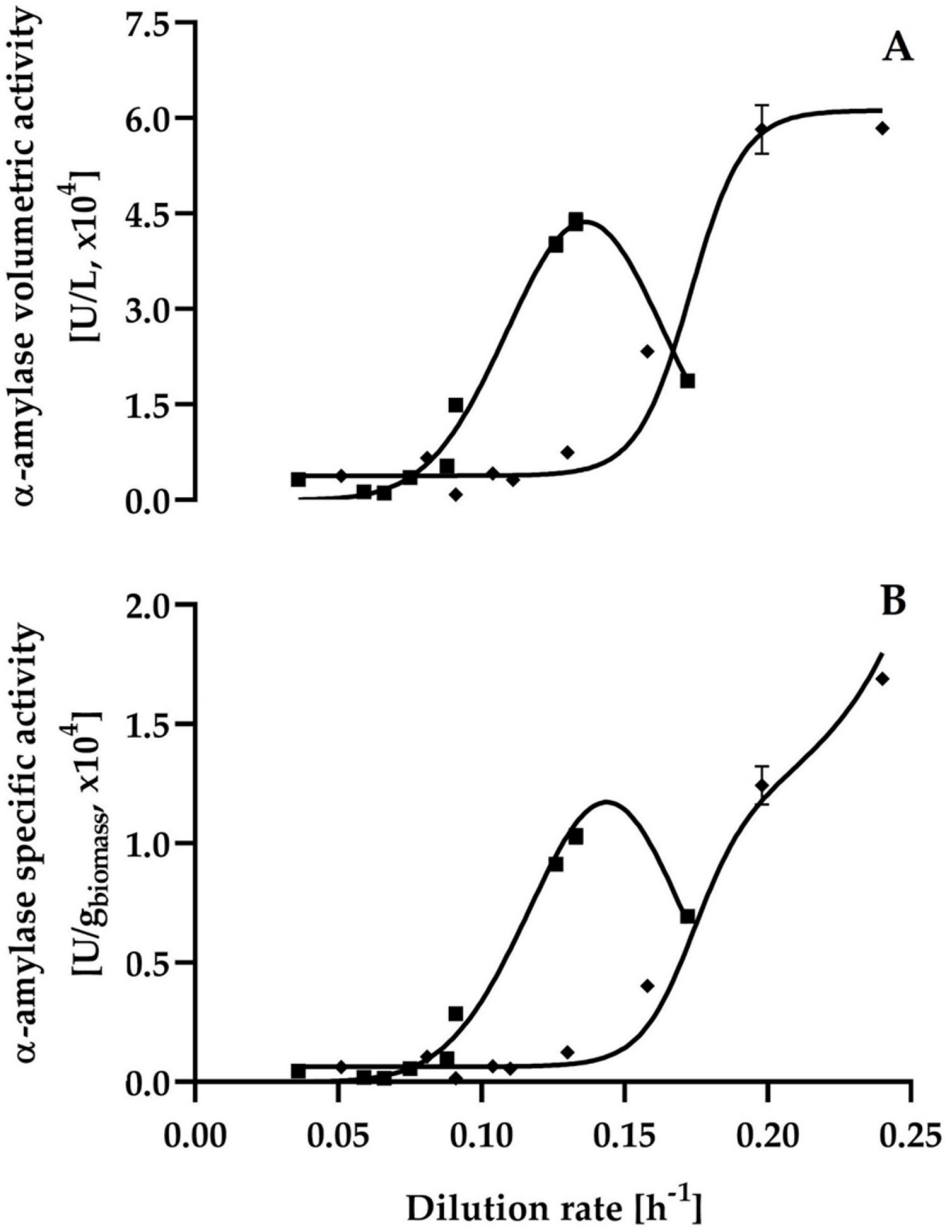
Steady-state α-amylase volumetric (A) and specific (B) activities versus dilution rate for continuous cultures of *C*. *wangnamkhiaoensis*. ◼, maltose; ◆, starch.

The growth and α-amylase production patterns of *C*. *wangnamkhiaoensis* (Figs 2A and 4) clearly suggest that α-amylase production and yeast biomass concentration are not directly related. However, the synthesis of the α-amylase is related to the type of carbon source present in the influent culture medium and to the dilution rate.

*C*. *wangnamkhiaoensis* was capable of producing high α-amylase activity when maltose and starch were used as carbon sources (Fig 4). However, no α-amylase activity was detected when glucose was used as the carbon source in the influent medium. These findings indicate that α-amylase synthesis of *C*. *wangnamkhiaoensis* is catabolically repressed by glucose and induced by maltose and starch.

The role of glucose in the production of yeast α-amylases is controversial. α-Amylase production is completely repressed by glucose in *Pichia burtonii* [30], *Candida tropicalis* CBS 6948 [31], and *Lipomyces starkeyi* CBS 1809 [32]. It is partially repressed by glucose in *Aureobasidium pullulans* N13d [33], *Schwanniomyces occidentalis* CSIR-Y828, *S*. *occidentalis* CSIR Y993, *Lipomyces kononenkoae* IGC 4052 [34], *L*. *starkeyi* 1807 [35], and *Schwanniomyces castelli* ATCC 26077 [36]. In contrast, no glucose repression of α-amylase was evident with *Torulopsis ingeniosa* Di Mena, *L*. *starkeyi* [37], *L*. *kononenkoae* 4052 B [34], *Dioszegia fristingensis* (T9Df1), *Dioszegia* sp., and *Leuconeusospora* sp. T11Cd2 [38].

It is well known that cells can adapt their gene expression and consequently their cell metabolism for optimal utilization of the carbon sources available in the environment. In bacteria, filamentous fungi, yeasts, and metazoa, the presence of glucose in the environment prevents the utilization of other available carbon sources by mechanisms known as glucose repression, or more generally, carbon catabolite repression [39]. Carbon catabolite repression is one of the main regulatory phenomena that helps microbial cells use available carbon sources in the most efficient way [40]; it also involves the regulation of a multitude of genes and proteins involved in carbon source utilization and energy generation [41].

In *Saccharomyces cerevisiae* or baker’s yeast, which has long served as a model for studying glucose repression, when glucose is present in the growth medium, uptake and catabolism of other carbon sources is repressed via three signaling pathways: inhibition of Snf1 protein kinase, activation of cAMP-dependent protein kinase A (PKA), and the regulation of transporter expression and stability at the plasma membrane by the yeast casein kinases Yck1 and Yck2 [39]. Although glucose repression has been extensively investigated, much still remains to be elucidated in order to achieve a full understanding of this regulatory mechanism.

In contrast, compounds composed of α-1,4-D-glucose units linked by α-D-(1–4) linkages such as maltose and starches, are inducers of α-amylase synthesis in several species of yeasts. These include *Cryptococcus gilvescens*, *Mrakia robertii* [38], *Pichia membranifaciens* [4], *Aureobasidium pullulans* N13d [33], *Pichia burtonii* [30], *Torulopsis ingeniosa* Di Mena [37], *Lipomyces starkeyi* [32,35], *Schwanniomyces castelli* [36,42], *Endomycopsis fibuligera* [42], *Candida tropicalis* CBS 6948 [31], *Schwanniomyces alluvius* ATCC 26074 [43], *Candida silvanorum*, *Candida tsukubaensis*, *Filobasidium capsuligenum*, *Leucosporidium capsuligenum*, and *Trichosporon pullulans* [44].

Our results clearly show that α-amylase synthesis of *C*. *wangnamkhiaoensis* is subject to induction by maltose and starch, as well as carbon catabolite repression by glucose. The potential for both induction and carbon catabolite repression in a cell ensures that inducible enzymes are produced in the correct proportions, only in the presence of the substrate and that neither valuable nutrients, energy nor amino acids are wasted in making unnecessary enzymes, but that when needed, these enzymes can be formed quickly [45].

The α-amylase volumetric (P) and specific (Y_px_) activities were found to have a sharp peak at a dilution rate of 0.13 h^-1^ (Fig 4) when maltose was used as a carbon source, with maximum α-amylase activity values of 43700 ± 1300 U/L and 10200 ± 300 U/g biomass, respectively (Table 1). This behavior has been interpreted to reflect a balance between induction and catabolite repression. At low dilution rates, the degree of enzyme induction by the substrate present in the chemostat limits the amount of the enzyme synthesized. In contrast, at dilution rates above the inflection point in the plots of enzyme specific activity versus dilution rate, the increase in the concentration of the substrate in the chemostat leads to a progressive increase in the catabolite repression of enzyme synthesis [15,46,47]. According to Lemmel et al. [29], catabolite repression of amylase synthesis has been observed in many carbon sources, including glucose, maltose, and starch at high concentrations.

**Table 1:** Kinetic parameters of α-amylase production by *C*. *wangnamkhiaoensis* in steady-state continuous cultures.

Parameter	Maltose	Starch
Maximum volumetric activity [U/L]	43700 ± 1300	58400 ± 800
Maximum specific activity [U/g biomass]	10200 ± 300	16900 ± 200
Maximum volumetric productivity [U/L·h]	5810 ± 170	14000 ± 200
Maximum specific productivity [U/g biomass·h]	1370 ± 40	4050 ± 60

In contrast, when soluble starch was used as a carbon source, the volumetric and specific activities of α-amylase were constant over the range of dilution rates from 0.05 to 0.11 h^-1^. However, at higher dilution rates (> 0.11 h^-1^), the activities increased progressively as the dilution rate increased (Fig 4), so that the highest enzyme activities were obtained at the highest dilution rate (0.24 h^-1^). The increase in the specific activity of an enzyme with an increase in the dilution rate is thought to be because the substrate or its metabolites present in the culture induce the synthesis of the enzyme and this induction remains partial at submaximal dilution rates [15,46].

The maximum levels of α-amylase volumetric and specific activity were higher in soluble starch (58400 ± 800 U/L and 16900 ± 200 U/g biomass) than in maltose-supplemented medium (43700 ± 1300 U/L and 10200 ± 300 U/g biomass). This indicates that starch is a better inducer than maltose for the synthesis of *C*. *wangnamkhiaoensis* α-amylase (Table 1).

**Fig 5:**
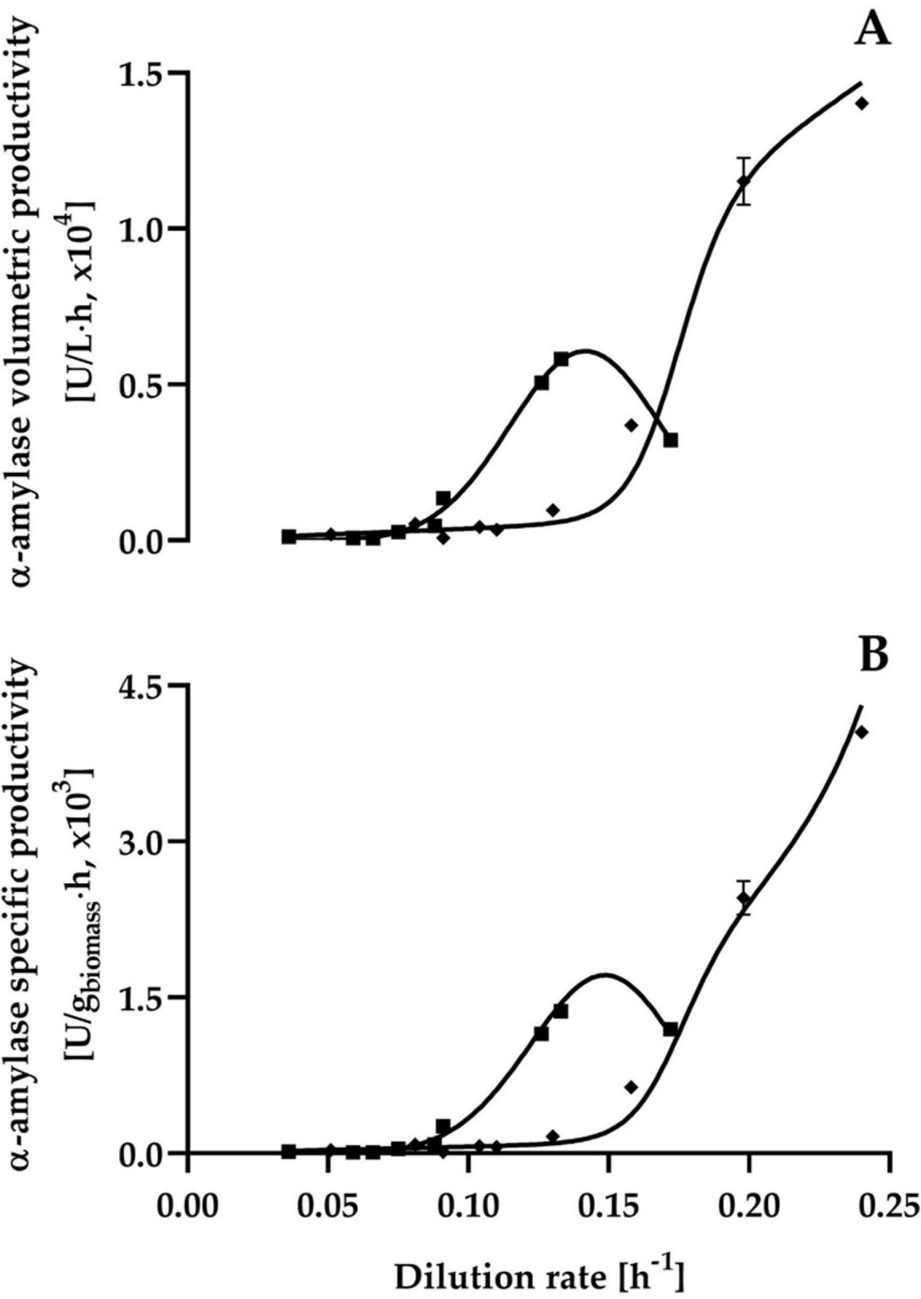
Steady-state α-amylase volumetric (A) and specific (B) productivities versus dilution rate for *C*. *wangnamkhiaoensis* continuous cultures. ◼, maltose; ◆, starch.

The results of α-amylase volumetric and specific productivities of *C*. *wangnamkhiaoensis* at the steady-state of single-stage continuous cultures are shown in Fig 5. When maltose was used as a carbon source, a peak of volumetric and specific productivity of α-amylase was obtained at a dilution rate of 0.133 h^-1^, with values of 5810 ± 170 U/L·h and 1370 ± 40 U/g biomass·h, respectively. In contrast, the volumetric and specific productivities of α-amylase were maintained practically constant in the range of dilution rates from 0.05 to 0.11 h^-1^. From this last dilution rate, both α-amylase productivities gradually increased as the dilution rate increased (Fig 5). Therefore, the maximum α-amylase volumetric and specific productivities were obtained at the highest dilution rate tested (0.24 h^-1^), with values of 14000 ± 200 U/L·h and 4050 ± 60 U/g biomass·h, respectively (Fig 5 and Table 1).

It is evident that the highest α-amylase productivities were obtained with starch as the carbon source, compared to maltose. This is economically advantageous due to the large availability of starch in nature.

The above results clearly demonstrated that the single-stage steady-state continuous culture is a satisfactory mode of operation of an airlift bioreactor, since it allowed high levels of production of yeast α-amylase. *C*. *wangnamkhiaoensis* is an attractive microbial source for α -amylase production.

## 4. Conclusions

This work studied the regulatory mechanisms of α-amylase synthesis and the growth and α-amylase production kinetics of *C*. *wangnamkhiaoensis* in single-stage steady-state continuous cultures in a split-cylinder internal-loop airlift bioreactor. Biomass yield was higher on glucose (0.535 ± 0.030 g/g) than on starch (0.491 ± 0.035 g/g) and maltose (0.456 ± 0.033 g/g); however, the maximum volumetric productivity of biomass was higher on starch (0.90 ± 0.016 g biomass/L·h) than on glucose (0.71 ± 0.036 g biomass/L·h) and maltose (0.55 ± 0.015 g biomass/L·h). No α-amylase production was observed when *C*. *wangnamkhiaoensis* was grown on glucose, suggesting that glucose catabolically repressed the synthesis of the enzyme. In contrast, maltose and starch induce the synthesis of high levels of α-amylase, with starch being the strongest inducer. The maximum volumetric activity, maximum specific activity, maximum volumetric productivity, and maximum specific productivity of α-amylase achieved with starch were 33.64 ± 3.37%, 65.69 ± 4.11%, 140.96 ± 6.08%, and 195.62 ± 7.43% higher respectively, than that obtained with maltose. These results are economically significant because starch is a cheap and profuse polysaccharide that can be hydrolyzed to simple sugars by *C*. *wangnamkhiaoensis* α-amylase and, subsequently, simple sugars can be used in the production of several high added-value compounds of industrial interest through fermentation processes.
